# Multiple Introductions of the Asian Longhorned Tick (*Haemaphysalis longicornis*) to the United States Revealed Using Mitogenomics

**DOI:** 10.1002/ece3.71312

**Published:** 2025-04-24

**Authors:** Zoe E. Narvaez, Andrea M. Egizi, Michael J. Yabsley, Alec T. Thompson, Mohamed Moustafa, Erika Alt, Matthew Bickerton, Kim Bjorgo, Rebecca A. Butler, Alexandra Cumbie, Gillian Eastwood, Richard C. Falco, Dina M. Fonseca, Jun Hang, Vanessa L. Harper, Nicole Lewis, Jan Lovy, Lauren P. Maestas, Thomas N. Mather, Ryo Nakao, James L. Occi, Tadhgh Rainey, Melanie Sal, Craig A. Stoops, Rebecca T. Trout‐Fryxell, Wes Watson, Nicole E. Wagner, Aihua Zheng, Perot Saelao, Dana C. Price

**Affiliations:** ^1^ Center for Vector Biology, Rutgers University New Brunswick New Jersey USA; ^2^ Tick‐Borne Diseases Laboratory Monmouth County Mosquito Control Division Tinton Falls New Jersey USA; ^3^ Southeastern Cooperative Wildlife Disease Study, College of Veterinary Medicine University of Georgia Athens Georgia USA; ^4^ United States Department of Agriculture National Bio & Agro‐Defense Facility Manhattan Kansas USA; ^5^ Animal Health Division West Virginia Department of Agriculture Charleston West Virginia USA; ^6^ Bergen County Department of Health Services Paramus New Jersey USA; ^7^ Department of Biology & Environmental Science West Virginia Wesleyan College Buckhannon West Virginia USA; ^8^ Department of Entomology & Plant Pathology University of Tennessee Knoxville Tennessee USA; ^9^ Department of Entomology Virginia Polytechnic Institute and State University Blacksburg Virginia USA; ^10^ New York State Department of Health, Vector Ecology Laboratory Fordham University Armonk New York USA; ^11^ Viral Diseases Program Walter Reed Army Institute of Research Silver Spring Maryland USA; ^12^ USDA Veterinary Sciences Seneca Rocks West Virginia USA; ^13^ Division of Animal Health New Jersey Department of Agriculture Ewing New Jersey USA; ^14^ Office of Fish and Wildlife Health and Forensics New Jersey Fish and Wildlife Oxford New Jersey USA; ^15^ Cattle Fever Tick Research Unit United States Department of Agriculture Agricultural Research Service Edinburg Texas USA; ^16^ TickEncounter Resource Center, University of Rhode Island Kingston Rhode Island USA; ^17^ Laboratory of Parasitology, Faculty of Veterinary Medicine Hokkaido University Sapporo Hokkaido Japan; ^18^ Hunterdon County Department of Health Flemington New Jersey USA; ^19^ Entomology, Environmental Health Section Defense Health Agency Brian D. Allgood Army Community Hospital Camp Humphreys Republic of Korea; ^20^ Department of Entomology & Plant Pathology North Carolina State University Raleigh North Carolina USA; ^21^ Institute of Zoology, Chinese Academy of Sciences Beijing China; ^22^ Veterinary Pest Genetics Research Unit United States Department of Agriculture Agricultural Research Service Kerrville Texas USA

**Keywords:** Asian longhorned tick, invasive species, livestock pest, mitogenome, population genetics

## Abstract

The Asian longhorned tick (ALT), *Haemaphysalis longicornis* , is a three‐host hard tick native to East Asia. Its opportunistic feeding habits make it an acute agricultural and medical threat, capable of spreading various zoonotic pathogens. An affinity for livestock and companion animals has allowed parthenogenetic populations of ALT to travel to and establish in overseas locations including the United States. To better understand the population dynamics of this rapidly expanding species, we sequenced the complete mitogenome of specimens collected from native and invasive ranges and performed phylogeographic analyses. As well as illustrating the diversity of Australasian and US ALT haplotypes, these methods have allowed us to estimate the source and frequency of successful introductions to the US. We highlight four potential introductions of parthenogenetic ALT, with likely origin populations identified in the Republic of Korea and Japan. These findings provide insight into potential routes of entry for ALT and other invasive tick species.

## Graphical abstract

The Asian longhorned tick, *Haemaphysalis longicornis,* is a significant global vector and threat to both agriculture and public health. Formerly known only in the Eastern Hemisphere, parthenogenetic ALT have been reported from 20 states across the eastern and central US, with a pattern of expansion suggestive of multiple distinct populations. Our mitogenomic analysis of specimens from both native and invasive populations provides evidence for the first time of at least four separate US introductions of parthenogenetic ALT and directly links each lineage to respective and putative source populations identified in East Asia.

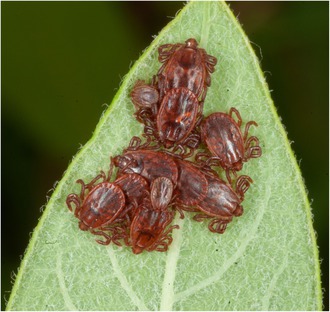

## 1 Introduction

Invasive ixodid ticks are responsible for billions of dollars of agricultural revenue losses each year in the form of animal morbidity, mortality, and management (Almazan et al. 2018). Livestock tick species, including *Rhipicephalus microplus* and *Rhipicephalus annulatus*, are notoriously difficult to control and contain, due in large part to the commercial transportation of their hosts (Giles et al. 2014). In the US, where the cattle industry is valued at over $80 billion (2023 total cash receipts; USDA ERS 2023), efforts to manage the spread of these livestock ticks involve intensive and coordinated actions by veterinary services and agriculture departments (Busch et al. 2014; Giles et al. 2014). Yet as international trade and travel increase, so do the long‐range movements of ectoparasites and associated pathogens.

*Haemaphysalis longicornis* Neumann (Ixodida: Ixodidae), the Asian longhorned tick (ALT), is uniquely poised for globalization. Native to China, Japan, the Republic of Korea (ROK), and parts of eastern Russia, this species exists in both bisexual and parthenogenetic reproductive lineages (Hoogstraal et al. 1968; Zhao et al. 2020). Parthenogenetic individuals are especially prolific, as a single unmated female is capable of producing up to 2000 eggs (Hoogstraal et al. 1968). Indeed, invasive parthenogenetic populations have long been established in parts of Oceania, including Australia, New Zealand, and various Pacific islands, and are thought to have been imported on cattle from Japan in the late nineteenth century (Hoogstraal et al. 1968).

An affinity for livestock and companion animals heightens the threat of ALT dispersal via long‐range import and export. Individuals have been intercepted several times on animals entering the United States, including a horse imported to New Jersey in 1969 (Burridge 2011) as well as on at least two horses in California during the 1980s (USDA APHIS 1982, 1983; Burridge 2011). In 1967, a female ALT was removed from an Australian sheepdog arriving in Hawaii, ultimately bound for Texas (Hoogstraal et al. 1968). The first evidence of successful establishment in the US was documented in New Jersey in 2017 when an Icelandic sheep was found to harbor all ALT life stages despite having no travel history (Rainey et al. 2018). Invasive ALT have since been reported in 21 US states, as far north as Massachusetts, as far south as Georgia, and as far west as Oklahoma (Yabsley and Thompson 2023; Cammack 2024; USDA APHIS 2024a). Further examination of archived specimens has revealed the presence of ALT in the US as early as 2010 (in West Virginia) (Beard et al. 2018). All lineages currently found in the US are believed to be parthenogenetic, as evidenced by a lack of confirmed field‐collected male specimens and the ability to continuously maintain reproductive colonies in the absence of male ticks.

In the Eastern Hemisphere, heavy ALT infestations of livestock hosts are associated with dangerous anemia and other sequelae associated with heavy tick feeding, often leading to animal death (Hoogstraal et al. 1968; Neilson and Mossman 1982; Yabsley and Thompson 2023). Anemia among infested livestock may also be attributed to the transmission of *Theileria orientalis*, the protozoan agent of bovine theileriosis. Most notably, the pathogenic *T. orientalis* Ikeda genotype, associated with substantial production losses in Asia, Australia, and New Zealand (Ota et al. 2009; Perera et al. 2014; Lawrence et al. 2021; Liu et al. 2022), has now been detected in 15 US states (USDA APHIS 2024b) and ALT have been linked to its transmission (Oakes et al. 2019; Dinkel et al. 2021). Should this pathogen continue to spread throughout the current US ALT range, it has the potential to impact states collectively responsible for over 20% of the US cattle inventory (USDA NASS 2021).

While not preferred hosts, humans are occasionally parasitized by ALT (Wormser et al. 2020; Bickerton and Toledo 2020). In Asia, these ticks vector *Dabie bandavirus* (formerly known as severe fever with thrombocytopenia syndrome virus [SFTSV]) as well as *Rickettsia japonica* , the agent of Japanese spotted fever (Zhao et al. 2020). The human vector potential of US ALT is not yet known; laboratory experiments have revealed it is a competent vector of *Rickettsia rickettsii* (Stanley et al. 2020) but detection of human pathogens in field‐collected ticks has been rare (Cumbie et al. 2022; Dye‐Braumuller et al. 2023). Many tick‐borne pathogens are maintained in small rodent reservoir hosts; however, ALT more frequently infest larger‐sized wildlife including woodchucks ( *Marmota monax* ), striped skunks ( *Mephitis mephitis* ), and white‐tailed deer ( *Odocoileus virginianus* ) (White et al. 2021; Levin et al. 2021; Ferreira et al. 2023).

Prior investigations of the phylogeographic structure of invasive ALT populations have demonstrated that US ALT belong to parthenogenetic lineages native to Asia (Egizi et al. 2020; Zhang et al. 2022). Through analysis of the mitochondrial *cox1* gene, Egizi et al. (2020) confirmed the introduction of at least three unrelated individuals, comprising three *cox1* haplotypes, to geographically distinct locations within the US. Since this initial investigation, ALT have been detected in nine additional US states. Habitat suitability models suggest a potential expanded range covering much of the eastern US as well as agriculturally significant parts of the northwestern and central US (Raghavan et al. 2019; Rochlin 2019; Namgyal et al. 2020; USDA NASS 2021).

Particularly given that ALT have the potential to establish in areas of the US with significant cattle production, we must prioritize the monitoring of populations in the continental US and prevent the introduction of additional tick and pathogen genotypes from abroad. Identifying the routes and means by which invasive species are introduced is crucial for the design of effective management and prevention strategies, specifically as they relate to the implementation of quarantine measures, import/export regulation, and public cooperation. Population source tracing can also be an important tool to identify locations for exploration of natural enemies to be used as bio‐control agents (Goolsby et al. 2015), as well as for examining population‐level variation in genotypes as it relates to differences in phenotypes such as vector competence, acaricide resistance, or hardiness to environmental factors such as hotter or colder climates (i.e., genotype/phenotype relationships) (Rodriguez‐Vivas et al. 2012; Vega‐Rúa et al. 2020; McGaughran et al. 2024). To these ends, we have conducted a phylogeographic analysis using a broad and diverse set of complete mitochondrial genomes generated from parthenogenetic and bisexual ALT populations spanning the Eastern and Western Hemispheres. Our results illustrate genetic variation between and within major ALT lineages while also identifying potential source populations of invasive North American ticks.

## 2 Materials and Methods

### 2.1 Tick Sample Sourcing

ALT specimens were sourced from several US states, including West Virginia (*n* = 5), Tennessee (*n* = 3), New Jersey (*n* = 4), Virginia (*n* = 3), North Carolina (*n* = 2), Rhode Island (*n* = 1), and Pennsylvania (*n* = 1), as well as the ROK (*n* = 3). Also included were a subset of banked DNA isolates extracted for *cox1* barcoding by Egizi et al. (2020), representing US states New Jersey (*n* = 13), West Virginia (*n* = 6), Delaware (*n* = 4), Virginia (*n* = 3), New York (*n* = 2), and Arkansas (*n* = 1), as well as Japan (*n* = 7), China (*n* = 2), the ROK (*n* = 2), and Australia (*n* = 1) (Table S1). In total, 48 US specimens and 15 overseas specimens were selected for whole‐mitogenome sequencing. Overseas specimens sequenced for this study were limited to known US‐invasive lineages (e.g., *cox1* haplotypes H1, H2, and H3, as defined in Egizi et al. 2020), except for two bisexual lineage ticks from China and the ROK, which were selected to supplement the outgroup. Specimens were collected between 2013 and 2023 either through environmental sampling (using drag or flag methods) or host sampling (Table S1). We also obtained three legs from a larval ALT collected from a white‐tailed deer in WV, USA, in 2010 from the USDA National Veterinary Services Laboratories to place the earliest known detection of this tick in our evolutionary analysis.

To increase representation of the Australasian range, we downloaded 110 additional ALT mitogenome sequences from NCBI GenBank (accessed March 2023) largely generated in the analysis of Zhang et al. (2022). Also included were sequences corresponding to accession numbers LC667696, LC667699, LC667702, LC667722, LC667729, LC667734, LC667735‐LC667737, LC667741, and LC667742. These mitogenomes represented specimens from China (*n* = 64), Japan (*n* = 35), Fiji (*n* = 4), Australia (*n* = 2), New Caledonia (*n* = 2), New Zealand (*n* = 1), and the ROK (*n* = 1) as well as a single specimen from the US (NJ). The downloaded sequences included some additional *cox1* diversity beyond the 3 haplotypes present in the US.

### 2.2 DNA Extraction and Mitochondrial Genome Sequencing

Whole specimens sourced for this project were extracted using Thermo Scientific GeneJET DNA Purification Kit (Thermo Fisher Scientific). Briefly, whole ticks were homogenized in 20 μL Digestion Solution (adult ticks were first bisected) with two 3‐mm stainless steel beads in a VWR Mini Bead Mill Homogenizer (VWR International) and extracted per manufacturer protocol with overnight lysis at 56°C. PCR Amplification of the ~14.7 kb mitochondrial genome was performed using the six primer pairs of Zhang et al. (2022), with each set of primers producing a roughly 3 kb amplicon. Each reaction consisted of 2 μL DNA template, 12.5 μL LongAmp Taq 2X Master Mix (New England Biolabs), 1 μL each primer (10 μM), and 8.5 μL ultrapure water for a final reaction volume of 25 μL. Amplification was carried out under the following reaction conditions: initial activation at 94°C for 2 min, 35 cycles of 94°C for 20 s, 50°C for 15 s, and 65°C for 2.5 min, followed by final extension at 65°C for 10 min. All products were visualized on a 1% agarose gel stained with Midori Green Xtra (Nippon Genetics), and successful amplifications were cleaned using the illustra ExoProStar kit (Cytiva). DNA was quantified using a Qubit dsDNA broad‐range kit (ThermoFisher Scientific). PCR products corresponding to each sample were brought to equimolar concentration and pooled. Illumina sequencing libraries were prepared using the Illumina DNA Sample Prep Kit (Illumina Inc.) and sequenced on the Illumina MiSeq instrument using a 500‐cycle sequencing kit in 250 × 250 bp paired‐end mode. Reads were quality/adapter trimmed with bbduk of the bbtools package (https://jgi.doe.gov/data‐and‐tools/software‐tools/bbtools/) and mapped to the mitochondrial genome generated from a parthenogenetic tick collected at the index site of the first reported US population (Zhang et al. 2022). The mappings were used to construct consensus sequences in Geneious Prime version 2023.2.1.

### 2.3 Haplotyping of Earliest Known ALT in US

Due to its importance as a voucher, the larval tick collected from WV in 2010 could not be extracted in its entirety. Three legs were removed and subjected to a modified extraction protocol based on the methods of Patzold et al. (2020). Briefly, the legs were incubated overnight at 56°C in 45 μL Buffer ATL (Qiagen) and 5 μL Proteinase K (Qiagen). The remainder of the extraction process followed the Oligonucleotide Clean‐up protocol of the Monarch PCR & DNA Clean‐up Kit (New England Biolabs), using 100 μL DNA Cleanup Binding Buffer, 300 μL ethanol, and 500 μL DNA Wash Buffer. Elution was performed in 17.5 μL Elution Buffer, with a 10 min incubation at room temperature; this step was repeated with an additional 15 μL Elution Buffer. Attempted whole‐mitogenome amplification of this sample was followed by amplification of two *cox1* fragments known to contain variable sites in US populations (Egizi et al. 2020).

The primers chelicerateRv (5′‐CCTCCTCCTGAAGGGTCAAAAAATGA‐3′, Barrett and Herbert 2005) and HchorCOIFint (5′‐CATTATGGGCCATCTGTAGATATAGC‐3′, designed by A. Egizi for unpublished work on *Haemaphysalis* spp.) were used to amplify a 214 bp piece differentiating H2 from H1/H3 (site where H2 has a G; H1/H3 both have Ts). The primers TypeF2 (5′‐ATTAGGAGCWCCWGATATAGC‐3′; Hernández‐Triana et al. 2014) and TypeR3 (5′‐GGTGGATAAACAGTTCAWCC‐3′; Hebert et al. 2013) were used to amplify a 93 bp piece differentiating H3 from H1/H2 (H3 has an A where H1/H2 have Gs). For both fragments, the 20 μL reaction volume contained 0.2 μL AmpliTaq Gold (Life Technologies), 0.5 μL each primer (10 μM), 2 μL 10X Buffer II (ThermoFisher Scientific), 2 μL MgCl_2_ (25 mM), 0.3 μL Bovine Serum Albumin (BSA; 10 mg/mL), 0.5 μL dNTP mix, 1 μL template DNA, and the remainder ultrapure water. PCR reaction conditions were as follows: an initial activation step of 95°C for 10 min, followed by 45 cycles of 95°C for 30 s, 50°C for 30 s, and 72°C for 45 s; with a final extension of 72°C for 7 min.

### 2.4 Phylogeny Reconstruction

Both the consensus mitochondrial genome sequences generated here and the downloaded NCBI references (total 102 parthenogenetic and 71 bisexual individuals) were aligned together using mafft‐einsi v. 7.4 (Katoh and Standley 2013). The alignment was then used to construct a maximum‐likelihood phylogeny using IQTREE v. 2.2 (Minh et al. 2020) under automatic model selection (K3Pu + F + I + G4 being the selected model), with nodal supports assessed via 2000 rapid bootstrap approximations.

### 2.5 Isolation by Distance and Principal Component Analyses

To assess population isolation by distance in both native and invasive ranges, we performed Mantel tests using matrices of pairwise raw genetic distance and Haversine geographic distance. Geographic coordinates were based upon the approximate center of the jurisdiction of collection, as we could not confirm the exact collection location of each specimen. Separate correlation tests were conducted for East Asian samples (China, Japan, and the ROK) and US samples. Tests were carried out in R 4.4.0 (R Core Team 2021), using function mantel.test of the *vegan* package, with 999 permutations. To visualize genetic diversity among US‐invasive lineages, we performed principal component analyses (PCA) using single nucleotide polymorphism (SNP) data from the clades corresponding to *cox1* haplotypes H1, H2, and H3. The R package *adegenet* version 2.1.1 (Jombart 2008) was used for analysis and visualization.

### 2.6 Selective Pressure Analysis

The diverse sample size assembled here afforded us the ability to explore individual mitochondrial loci that may be under selection as parthenogenetic and bisexual lineages continue to diverge, and further as parthenogenetic lineages expand globally. Amino acid translations for each gene were excised from the mitochondrial alignment using Geneious Prime, and codeml of the PAML package (Yang 2007) was used to infer selective coefficients (i.e., omega [*ω*]) values on branches of the phylogeny under the branch model. We tested the basal branch connecting the parthenogenetic and bisexual lineages, and each of the three individual branches comprising the H1, H2, and H3 lineages. The latter three tests used a phylogeny that did not contain the bisexual clade such that the null model M0 (one *ω* ratio for all sites) was calculated only for parthenogenetic ticks.

## 3 Results

### 3.1 Features and Diversity of Selected Mitogenomes

We generated an average of 1057‐fold sequencing depth for each ~14.7 kb mitochondrial genome in our analysis (minimum = 436, maximum = 3185). The 173 mitogenomes included in phylogenetic analysis—102 parthenogenetic and 71 bisexual—were alike in gene content and arrangement, with 13 canonical protein coding genes (PCGs), 2 rRNA genes, and 22 tRNA genes. Within the entire parthenogenetic lineage, the average identity of mitogenomic sequences was 99.82%. Among individual genes, including concatenated tRNAs, the highest genetic diversity (by % parsimony‐informative sites) was present in *atp8*, *cox3*, and *nad2*. The average number of haplotypes per gene was 11.1, with the greatest number of haplotypes found in *nad4*, tRNA, and *cox1* genes (Table 1, Table S2). Among the 49 US‐sourced sequences, average identity was 99.86%. The average number of haplotypes per gene was 2.6, with the greatest number of haplotypes recovered among concatenated tRNAs (5 haplotypes), *cox3* (4 haplotypes), and *nad5* (4 haplotypes) (Table 1, Table S2).

**TABLE 1:** Summary of variation in mitochondrial genes of all parthenogenetic specimens in this analysis (“Parth.”) and the subset of those sourced from within the United States (“Parth. [US]”).

Gene	Lineage	Reference nucleotide position	Length	Identical sites	% Polymorphic sites	Number of haplotypes	Parsimony informative sites	% Parsimony informative sites
cox1	Parth.	1121‐2659	1539	1523	0.0104	16	5	0.00325
cox1	Parth. (US)	1121‐2659	1539	1537	0.0013	3	2	0.0013
cox2	Parth.	2682‐3338	657	654	0.00457	4	1	0.00152
cox2	Parth. (US)	2682‐3338	657	657	0	1	0	0
cox3	Parth.	4292‐5068	777	759	0.02317	12	8	0.0103
cox3	Parth. (US)	4292‐5068	777	771	0.00772	4	5	0.00644
cytb	Parth.	13053‐14132	1080	1063	0.01574	14	7	0.00648
cytb	Parth. (US)	13053‐14132	1080	1074	0.00556	2	6	0.00556
nad1	Parth.	6716‐5769	948	939	0.00949	7	1	0.00105
nad1	Parth. (US)	6716‐5769	948	947	0.00105	2	1	0.00105
nad2	Parth.	82‐1008	927	911	0.01726	13	9	0.00971
nad2	Parth. (US)	82‐1008	927	923	0.00431	2	4	0.00431
nad3	Parth.	5140‐5466	327	319	0.02446	8	2	0.00612
nad3	Parth. (US)	5140‐5466	327	325	0.00612	3	2	0.00612
nad4	Parth.	12257‐10917	1341	1321	0.01491	18	8	0.00597
nad4	Parth. (US)	12257‐10917	1341	1336	0.00373	2	5	0.00373
nad4l	Parth.	12501‐12226	276	272	0.01449	5	2	0.00725
nad4l	Parth. (US)	12501‐12226	276	274	0.00725	3	2	0.00725
nad5	Parth.	10881‐9226	1656	1631	0.0151	15	13	0.00785
nad5	Parth. (US)	10881‐9226	1656	1650	0.00362	4	6	0.00362
nad6	Parth.	12638‐13082	445	441	0.00899	5	1	0.00225
nad6	Parth. (US)	12638‐13082	445	444	0.00225	2	1	0.00225
atp6	Parth.	3620‐4285	666	651	0.02252	15	6	0.00901
atp6	Parth. (US)	3620‐4285	666	665	0.0015	2	1	0.0015
atp8	Parth.	3471‐3626	156	154	0.01282	3	2	0.01282
atp8	Parth. (US)	3471‐3626	156	155	0.00641	2	1	0.00641
12SrRNA	Parth.	8666‐8023	643	629	0.02177	13	3	0.00467
12SrRNA	Parth. (US)	8666‐8023	643	641	0.00311	3	2	0.00311
16SrRNA	Parth.	7958‐6787	1173	1152	0.0179	12	5	0.00426
16SrRNA	Parth. (US)	7958‐6787	1173	1172	0.00085	2	1	0.00085
tRNAs	Parth.	NA	1342	1326	0.01192	18	8	0.00596
tRNAs	Parth. (US)	NA	1342	1338	0.00298	5	4	0.00298

### 3.2 Phylogenetic Analysis

**FIGURE 1:**
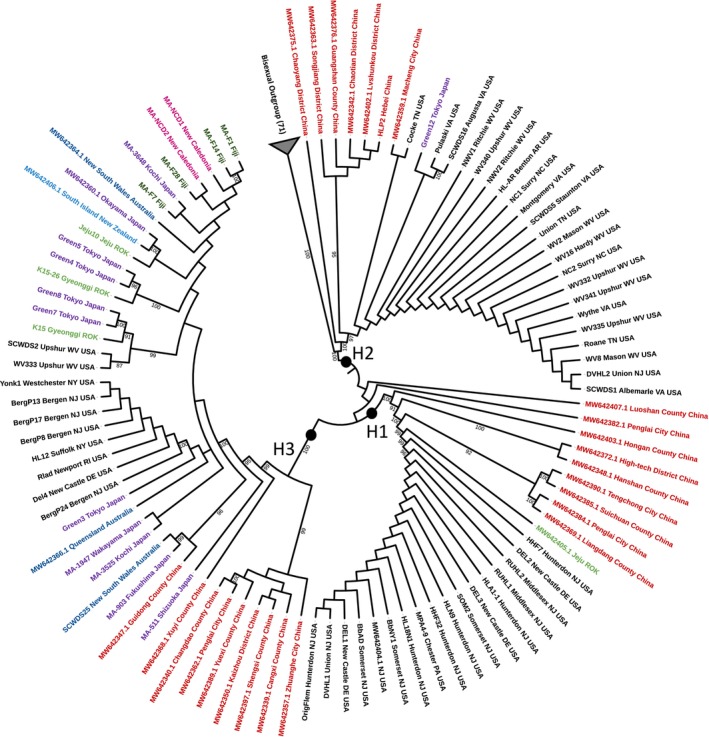
Maximum‐likelihood radial cladogram (branch lengths disabled) illustrating phylogenic relationships among parthenogenetic US and Australasian *H. longicornis* . The bisexual clade, rooting the tree, is collapsed. Specimen country of origin is distinguished by label color, with specimens from the US displayed in black. Only bootstraps > 70 are displayed. Note that all H1 ticks sourced from the US are identical at the sequence level.

The maximum‐likelihood phylogeny illustrated clear separation between parthenogenetic and bisexual clades with 100% bootstrap support (Figure 1). The parthenogenetic clade further assorted into three strongly supported monophyletic lineages, corresponding with the clades defined by *cox1* haplotypes H1, H2, and H3 of Egizi et al. (2020). This was expected, as the majority of specimens included in this analysis were selected on the basis of their *cox1* haplotype, with preference given to known US‐invasive lineages. Of note, some overseas specimens did constitute additional *cox1* haplotypes (16 haplotypes total). However, all specimens fell into the clades defined by the two polymorphic sites originally used to discriminate H1, H2, and H3 by Egizi et al. (2020). We will henceforth refer to the three mitochondrial lineages defined originally based on *cox1* haplotype but supported by full mitosequencing as clades H1, H2, and H3.

Each of these three major lineages contained both US‐sourced sequences and Australasia‐sourced sequences. The clustering of these sequences within these monophyletic lineages supports the introduction of at least three individual parthenogenetic ticks to the US. All US‐sourced ticks with *cox1* haplotype H1 were 100% identical across the mitochondrial genome (note that limited phylogenetic signal within the US H1 clade is the result of small gaps in sequencing coverage (< 30 bp) of specimen HHF7). Interestingly, the individual most closely grouped with H1 US ticks was collected on Jeju Island, ROK. All other current members of the H1 clade were sourced from China. Within the H2 lineage, the majority (19/22) of US‐sourced ticks formed a clonal clade. There was strong support for the clustering of samples from the US, China, and Japan. A specimen from Chaotian District, China, and a specimen from Tokyo, Japan, were genetically equidistant (99.993% identical) to the 19 clonal US specimens. Outside of the clonal US H2 clade, two H2 specimens from VA, USA, clustered together based on a single adenine to guanine SNP in the tRNA (trnL2) gene, located in the stem of the D arm. This mutation appears to have arisen independently within all three lineages, suggesting a highly variable locus. Additionally, the H2 sample from Cocke, TN, fell outside of the clonal US clade. This was due to the presence of a single SNP in the *cox3* region not shared by any other H2 sequences. The US‐sourced sequences within the H3 clade were polyphyletic– those originating in the northeastern US formed a clonal clade, while two specimens from WV were clearly excluded. The former clade was most genetically similar to a specimen from New South Wales, Australia (99.986% identical). The WV specimens, identical to one another, differed from the larger clonal US clade at 3 loci. These two specimens clustered with specimens from Japan and ROK with very high bootstrap support (96%). They shared a particularly close relationship with a single specimen sourced from Gyeonggi, ROK (99.993% identical). This topology within US H3 suggests two separate introductions within the broader H3 lineage.

### 3.3 Mantel Test for Isolation by Distance

**FIGURE 2:**
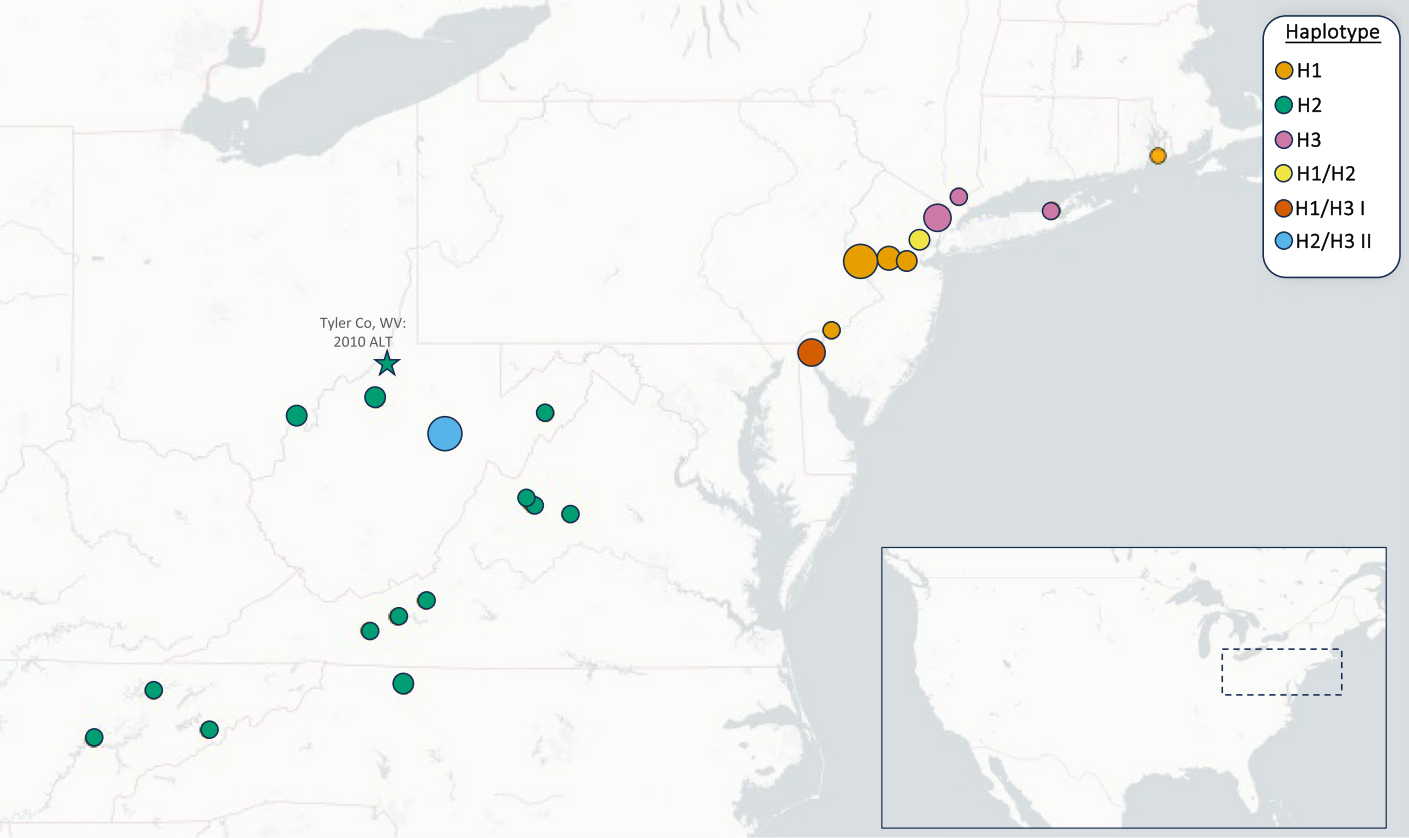
Distribution of the US‐invasive *H. longicornis* specimens sequenced for this analysis, with markers indicating the lineage (H1, H2, H3 I, and H3 II) or combination of lineages found in underlying counties. Note that marker size is scaled to represent number of specimens (minimum one, maximum six) sourced from each county. Not pictured: Single H2 specimen from Benton County, AR.

Our isolation by distance (IBD) analysis of East Asian specimens belonging to all three clades (H1, H2, and H3) indicated no significant correlation between genetic and geographic distance (r statistic = 6.733e‐5, *p* = 0.454). In contrast, analysis of US specimens yielded a highly significant result (*r* statistic = 0.515, *p* = 0.001). Figure 2 illustrates the geographic distribution of the US specimens included in this analysis.

### 3.4 Principal Component Analyses

**FIGURE 3:**
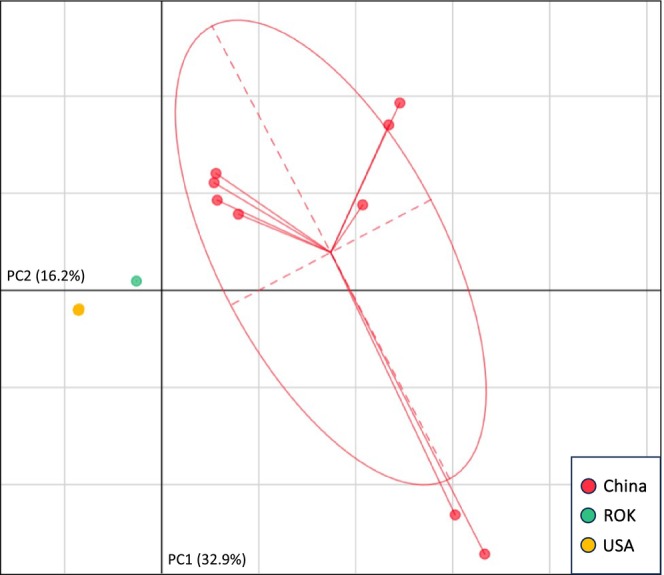
PCA illustrating mitogenomic diversity among H1 *H. longicornis* specimens only. Axes correspond to the first two components, PC1 and PC2. A 95% confidence ellipse is displayed for source countries with > 2 mitotypes. Note that single yellow point represents 17 identical USA samples. Country abbreviations: Republic of Korea (ROK), United States of America (USA).

**FIGURE 4:**
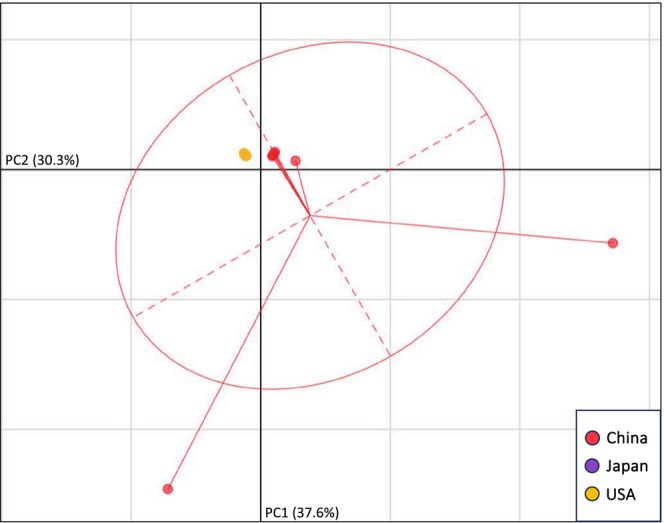
PCA illustrating mitogenomic diversity among H2 *H. longicornis* specimens only. Axes correspond to the first two components, PC1 and PC2. A 95% confidence ellipse is displayed for source countries with > 3 mitotypes. Note that the single H2 specimen from Japan is not visible due to overlap of USA specimens. Country abbreviations: United States of America (USA).

**FIGURE 5:**
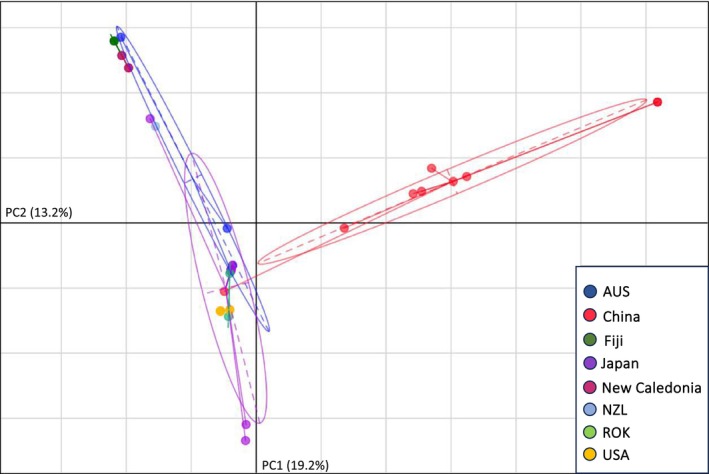
PCA illustrating mitogenomic diversity among H3 *H. longicornis* specimens only. Axes correspond to the first two components, PC1 and PC2. A 95% confidence ellipse is displayed for source countries with > 2 mitotypes. Note that the three specimens with the greatest genetic distance from US specimens (representing Japan and the ROK) have been removed. Country abbreviations: Australia (AUS), New Zealand (NZL), Republic of Korea (ROK), and United States of America (USA).

PCA of each major clade (H1, H2, and H3) allowed us to visualize mitogenomic similarity between US and Australasian ALT outside of a strictly bifurcating phylogeny. The PCA of H1 specimens (Figure 3) indicated a strong distinction between specimens from China versus those from the US and the ROK. Such a disparity was absent in the H2 PCA, where all US specimens (as well as the specimen from Japan) fell within the 95% confidence ellipse corresponding to Chinese specimens (Figure 4). The H3 PCA again suggested a disparity between China versus the US, Japan, the ROK, and Oceania (Figure 5). Notably, there were two distinct phylogenetic lineages of US H3. Both US clusters fell within the confidence ellipse corresponding to Japan, but outside of that corresponding to Australia.

### 3.5 Selective Pressure Analysis

Our initial test for differential selective pressure on mitochondrial PCGs between bisexual and parthenogenetic lineages produced a significant likelihood‐ratio test (LRT) (*χ* ^2^ = 0.0320) only for *nad4l*. This was driven by a single G → A mutation at nucleotide 115 in the alignment resulting in a V → M amino acid change, coupled with a lack of any non‐synonymous substitutions (NSSs) within the parthenogenetic lineage. Given this evidence for potential lineage‐specific variation in selective pressure, we proceeded to test each US‐invasive lineage individually, in the absence of the bisexual clade. A significant LRT was obtained only for the H1 lineage (as the foreground branch) in the nad1 gene (*χ* ^2^ = 0.0232), driven largely by a NSS in the subterminal codon leading to a F → Y amino acid change.

### 3.6 Haplotyping of Earliest Known ALT in US

Whole‐mitogenome amplification of the 2010 voucher specimen from Tyler County, WV was unsuccessful, likely due to excessive DNA fragmentation. However, PCR amplification of two relevant *cox1* fragments assigned this tick to the H2 haplotype. This confirms that the H2 lineage has been present in the US for at least a decade, representing the first known invasive lineage. This finding is consistent with the broad distribution of H2 ALT in the US, suggesting early arrival and dispersal (Figure 2).

## 4 Discussion

Our expanded mitogenomic analysis of ALT confirms the presence of three major lineages within the US as suggested by Egizi et al. (2020). Within each of these three monophyletic clades, US specimens are clustered with conspecifics from the Eastern Hemisphere, supporting the origination of each parthenogenetic lineage from distinct foreign populations. The clades are named based on the corresponding US *cox1* haplotype (H1, H2, and H3).

Of the potential source countries sampled, China and the ROK were found to harbor all three clades. Either of these nations could represent a parsimonious route of introduction, involving a single host bearing ALT from multiple lineages. However, this [a single introduction scenario] seems unlikely given the diverse phylogeographic relationships indicated by maximum‐likelihood analysis. Within each major parthenogenetic clade, US specimens cluster with specimens from different source countries. In the H3 clade alone, clustering of US specimens suggests at least two separate origins for invasive ticks. These putative H3 sources include Japan and Australia, countries not known to host all three major haplogroups currently found in the US. Further, the spatial clustering of US lineages, supported by significant IBD, is consistent with (though not conclusively demonstrative of) multiple introductions followed by localized spread.

In light of this evidence, we will discuss each mitochondrial clade separately under the assumption that it represents at least one isolated introduction of ALT to the US.

We found complete sequence identity among the mitogenomes of H1 ticks from the US. This strongly suggests that all members of this lineage are descended from a single individual, likely introduced to the northeastern part of the country. It is known that the H1 clade has been present in this region since at least 2013, when a single specimen was recovered from a dog in NJ (retroactively identified as ALT) (Egizi et al. 2020). There appears to have been relatively little diaspora over the past decade– the presently known distribution of the H1 lineage remains largely focused in the northeastern/mid‐Atlantic states of NJ, NY, PA, and DE (Figure 2).

The vast majority of Asian H1 specimens included in this analysis were of Chinese origin. Egizi et al. first noted the dominance of the H1 haplotype in China, also finding the lineages most closely related to H1 to be restricted to China and the ROK. Our dataset, with the inclusion of sequences published by Zhang et al. (2022), did include a single H1 tick from Jeju Island, ROK. Interestingly, this was the native‐range specimen most similar in sequence to US H1 ALT (99.986% identity; 2 SNPs). This raises the possibility of the US‐invasive H1 clade originating in the ROK rather than in China. Granted, we cannot ascertain that the individual H1 tick from Jeju represents a greater H1 population on the island. Jeju is a common stopover site for migratory birds, some of which are known hosts of ALT (Choi et al. 2014; Yun et al. 2015). However, we do have evidence that the specimen is substantially diverged from the clade of H1 specimens found in China (Figure 3). As the H1 clade is not known outside of China and the ROK, it is circumstantially likely that this US‐invasive lineage originated in the ROK.

Based on current data, the H2 clade is the most geographically expansive in the US. Known specimens range from central NJ to western AR (Figure 2). This degree of dispersal is suggestive of an earlier introduction relative to the other two US‐invasive clades. Indeed, based on the barcode of the archived larva from Tyler County, WV, the H2 lineage has been in the US since at least 2010. It is unknown whether WV was the initial site of establishment, but the current distributional extent of H2 is consistent with establishment and radiation from the mideastern US region. Populations of H2 ticks are widespread in Australasia, and members have been confirmed across East Asia (China, Japan, and ROK), as well as in Australia (Egizi et al. 2020). The clonal US‐sourced ticks included in our analysis were genetically equidistant to specimens from central China (Sichuan province) and Japan, though it should be noted that Australian H2 was not represented in the phylogeny.

While most US H2 ticks were identical, there were several individuals with slight genetic variation. Two specimens collected from Pulaski and Augusta counties in VA contained a SNP in the tRNA(Leu) gene, located in the D‐stem. This adenine to guanine modification would result in a non‐canonical base pairing between guanine and uracil, a phenomenon relatively common in the primary structure of tRNAs (Seelam et al. 2017). While unique within the H2 clade, this mutation was found to have arisen independently also in the H1, H3, and bisexual clades. Additionally, a single specimen from Cocke County, TN had a silent mutation in the *cox3* gene. This was the only noted member of the H2 clade with this polymorphism, though it has also arisen within H3. These independent occurrences suggest that both aforementioned sites are highly polymorphic.

Two geographic foci of US H3 ticks were represented in our dataset– one in the Northeast (NJ, NY, DE, and RI) and one in WV. Interestingly, these spatial clusters were genetically distinct as well. The northeastern group formed a clonal clade most similar to specimens from Australia and Japan, while the two specimens from WV were closely related to ticks sourced from Japan and the ROK. Particularly given the high bootstrap (99) clustering of WV with East Asia, we find it unlikely that either of the two H3 US clades are descended directly from the other. Instead, the polyphyly of US specimens suggests two separate introductions of H3 ALT, at least one of which likely originated in East Asia.

Also apparent in the phylogenetic arrangement of the H3 clade is the close relationship between samples from Japan and Oceania. That matches expectations since the parthenogenetic populations currently found throughout Oceania are thought to have arrived on cattle imported to Australia from Japan during the late nineteenth century (Hoogstraal et al. 1968). This relationship somewhat complicates any phylogeographic signal between Japan, Oceania, and the US. For instance, the clonal US H3 clade is most genetically similar to a tick from Australia (99.986% identical), but only marginally close relatives are also found in Japan (99.980% identity). Further, Oceania hosts a high frequency of ALT within the H4 lineage, a clade not known in the US (Egizi et al. 2020). Based on this frequency, an introduction event from Australia or New Zealand would have a high likelihood of involving H4 ticks. It thus remains possible that the H3 ticks presently found in the northeastern US originated in Japan rather than Australia, despite a close phylogenetic relationship with the latter. The H3 clade representatives from WV appear more certainly linked to East Asia, as these individuals cluster closely with specimens from both Japan and the ROK.

There are several potential mechanisms by which ALT could have entered the US, including on livestock, companion animals, or imported animal products (Burridge 2011). Notably, all but one documented interception of imported ALT has been associated with horses (Burridge 2011). This may reflect the stringency of equine importation requirements: horses arriving in the US are subject to quarantine and full‐body inspection for ectoparasites (USDA APHIS 2025). In general, livestock importation is subject to extensive government regulation; this oversight does not necessarily extend to the transport of companion animals. As postulated in Egizi et al., ALT could have arrived in the US on rescued dogs. Various US‐based organizations import large groups of dogs that have been removed from Korean dog meat farms, ultimately seeking to rehome them (Voorhees et al. 2017). In at least one known instance, these dogs were relocated to shelters in the NY/NJ area, where the H1 lineage appears concentrated (Egizi et al. 2020). All stages of ALT, including larvae, are found on stray and rural‐dwelling dogs in the ROK (Choe et al. 2011; Seo et al. 2020). Yet given the ROK's status as a low‐risk rabies country, the Centers for Disease Control and Prevention (CDC) does not currently require documentation of veterinary examination nor quarantine (CDC 2024). The import of dogs for adoption is subject to regulation by the Animal Welfare Act, although this legislation also does not stipulate examination/treatment for ectoparasites nor quarantine upon arrival in the US (USDA APHIS 2024c).

Understanding potential routes of past entry is critical to prevent future introductions, as well as to unravel the medical and veterinary risk of current US lineages. Across a broad Eastern Hemispheric range, ALT source populations may be differentially exposed to pathogens, pesticides, and abiotic pressures. While relatively little is known about potential ecogeographical variation among major parthenogenetic *H. longicornis* lineages, there is likely some degree of diversity in vector potential (Liu et al. 2023). Of note, *Dabie bandavirus* (SFTSV) is currently reported only from the East Asian portion of the *H. longicornis* range. The virus is endemic to China, Japan, and the ROK, where reports of minimum infection rates in adult ALT are as high as 6.5% (Zhang et al. 2022; Kim et al. 2023). The demonstrated transovarial transmission of this pathogen may elevate the risk of successful range expansion, eliminating the immediate need for a reservoir host (Casel et al. 2021). The potential spread of human pathogens underscores the need to stem further introductions of ALT.

Our knowledge of ALT source populations may allow us not only to implement policies that aim to prevent future introductions, but to more effectively control existing infestations. In anticipation of threats posed to livestock production, there have been several investigations into the chemical susceptibilities of ALT in the US (Bickerton et al. 2021, 2023; Butler et al. 2021). For the purpose of standardization, these trials generally source all experimental ticks from a single population or region. However, there may be potential for variation in acaricide susceptibility among lineages of US‐invasive ALT. As an example, the aforementioned island of Jeju, ROK, is the site of heavy pesticide use, with an average of 10,000 tons applied on the island annually (Park et al. 2020). An evaluation of ALT from Jeju found mortality to require a cypermethrin concentration two to seven times higher than the recommended dose (MJ 2014). These results highlight the possibility of differential chemical exposure among US‐invasive populations. Future investigations of acaricide susceptibility may benefit from inclusion and comparison of multiple invasive lineages.

Given the broad geographic range of ALT, there may also exist regional differences in physiological tolerance. Protein‐coding mitochondrial genes, responsible for oxidative phosphorylation and energy production, may be involved in environmental adaptation (Lubawy et al. 2022). While the order and sequence of these genes is relatively conserved, it has been demonstrated that mitogenomes of conspecific arthropod and other metazoan lineages may be subject to selective pressure, particularly driven by climatic stress (Camus et al. 2017; Li et al. 2018; Noll et al. 2022). We searched for potential targets of selection commensurate with diversification of major ALT lineages within our mitochondrial genomes and identified *nad4L* (NADH–ubiquinone oxidoreductase chain 4 L; electron transport from NADH to the respiratory chain) and *nad1* (NADH–ubiquinone oxidoreductase chain 1) as having potentially relevant amino acid substitutions in the parthenogenetic and H1 lineages, respectively. In both instances, the alignment shows a single NSS as differentiating the noted tick lineages; although only a single site, our analysis comprises closely related conspecific ticks from a broad geographic sampling, and it is thus likely that these polymorphisms are fixed in the respective populations that is, tick lineages as defined here. While these results do not necessarily indicate lineage‐specific adaptation, they do raise the possibility of differential selective pressure between ALT clades; *nad1* in particular was also identified as being under selection in divergent clades of gentoo penguins (Noll et al. 2022).

As such, future investigations into the adaptive potential of ALT may seek to differentiate between US‐invasive lineages. The traditional *cox1* barcoding locus remains a high‐resolution marker, allowing discrimination between the three major lineages currently present in the US. However, this locus fails to distinguish between the two separate H3 introductions evidenced here. Among the remaining mitochondrial PCGs, we found *nad5* to be most concisely informative of invasion lineage; this gene contains a 119 bp region with four distinct haplotypes, each characteristic of a separate introduction.

There are some limitations to our phylogeographic and selective pressure analyses. The sequenced specimens were sourced based on availability, representing varying collection methods and sample sizes. Any interpretations of the resulting phylogeography should therefore consider the potential for specimen migration. Ticks may leave their source populations on traveling hosts, moving over short or long distances depending on feeding duration and host behavior. As mentioned previously, ALT are known to parasitize a variety of migratory bird species, several of which migrate between China, Japan, and the ROK (Yun et al. 2015). While the potential for passive dispersal in North America remains to be seen, specimens have recently been removed from several migratory passerine species (Pandey et al. 2022). Some dispersal may then be anticipated along North American avian flyways, which generally follow a north–south pattern (Buhnerkempe et al. 2016). Mammalian hosts may also facilitate dispersal, particularly through human‐mediated transport. Livestock hosts such as cattle and sheep are frequently relocated across state lines and may be moved to different regions of the country for slaughter or sale (USDA ERS 2003; Cabezas et al. 2021).

Additionally, it is important to note that the Australasian samples included in this analysis were selected to represent US‐invasive lineages (on the basis of *cox1* sequence). This dataset is therefore not intended to capture the diversity of parthenogenetic ALT in Asia and Oceania. As illustrated by Egizi et al., there are numerous other *cox1* haplotypes present in this range, representing lineages not currently known in the US. These populations may have novel attributes, such as immunological competence and/or acaricide susceptibility (Liu et al. 2023). Through examining potential past routes of entry, we aim to prevent introductions of additional ALT populations to the US.

Finally, as in any examination of mitochondrial DNA, our analysis relies on certain assumptions of inheritance. We note that there is some record of heteroplasmy in ixodid ticks (Mastrantonio et al. 2019; Nadolny et al. 2015; Xiong et al. 2013), as well as suggestions of recombination in chelicerate mitogenomes (Gantenbein et al. 2005; Ovchinnikov and Masta 2012; Shao et al. 2005).

If present in ALT, these processes would conceivably represent sources of molecular evolution acting on the mitochondrion of bisexual populations that are absent in parthenogenetic ticks.

Parthenogenetic populations of ALT seem uniquely suited to disperse and thrive in our increasingly globalized world. Since the findings of Egizi et al. (2020), we have witnessed this vector proliferate across the eastern US, at the mounting expense of the livestock industry. Through this expanded phylogeographic analysis, we hope to better understand how ALT have reached the Western Hemisphere and how they may continue to disperse in the future. Chiefly, we emphasize the number and nature of introductions from abroad in hopes of preventing additional invasions. We demonstrate the existence of at least four founding populations, likely representing four separate introduction events. Phylogenetic relationships suggest distinct geographic origins for these founders, with most potential sources in East Asia.

As our world grows more interconnected, and international movement more common, we must be increasingly vigilant of hitchhiking ectoparasites. Globalization demands the rapid evolution of biosecurity strategies, based on a thorough understanding of diverse pests and pathogens. These measures must apply not only to agricultural trade, but to the travel of companion animals. Fortunately, the US has taken recent strides to prevent the import of parasites with canines. The Healthy Dog Importation Act, introduced in the US Senate in 2023, would require a certificate of veterinary inspection for any dog entering the country (Healthy Dog Importation Act 2023). While the passage of this bill should strengthen our defenses against exotic ectoparasite introductions, we must simultaneously monitor the movements of established US populations. As mentioned previously, the current US range of ALT spans only a portion of the projected suitable habitat in North America. While we may not be able to prevent the eventual expansion of the ALT range, we may temper its impact on animal and human health. We recommend targeted surveillance of ALT and sympatric pathogens, both within and outside of the currently known range.

## Author Contributions

**Zoe E. Narvaez:** conceptualization (equal), formal analysis (lead), investigation (lead), methodology (equal), project administration (equal), software (equal), validation (equal), visualization (equal), writing – original draft (equal), writing – review and editing (equal). **Andrea M. Egizi:** conceptualization (equal), data curation (equal), investigation (equal), methodology (equal), resources (equal), writing – original draft (equal), writing – review and editing (equal). **Michael J. Yabsley:** investigation (equal), resources (equal), writing – review and editing (equal). **Alec T. Thompson:** investigation (equal), resources (equal), writing – review and editing (equal). **Mohamed Moustafa:** data curation (equal), investigation (equal), resources (equal), writing – original draft (equal), writing – review and editing (equal). **Erika Alt:** resources (equal), writing – original draft (equal). **Matthew Bickerton:** resources (equal), writing – original draft (equal). **Kim Bjorgo:** resources (equal), writing – original draft (equal). **Rebecca A. Butler:** resources (equal), writing – original draft (equal). **Alexandra Cumbie:** resources (equal), writing – original draft (equal). **Gillian Eastwood:** resources (equal), writing – original draft (equal). **Richard C. Falco:** resources (equal), writing – original draft (equal). **Dina M. Fonseca:** conceptualization (equal), methodology (equal), resources (equal), writing – original draft (equal), writing – review and editing (equal). **Jun Hang:** resources (equal), writing – original draft (equal). **Vanessa L. Harper:** resources (equal), writing – original draft (equal). **Nicole Lewis:** resources (equal), writing – original draft (equal). **Jan Lovy:** resources (equal), writing – original draft (equal). **Lauren P. Maestas:** resources (equal), writing – original draft (equal). **Thomas N. Mather:** resources (equal), writing – original draft (equal). **Ryo Nakao:** resources (equal), writing – original draft (equal). **James L. Occi:** resources (equal), writing – original draft (equal). **Tadhgh Rainey:** resources (equal), writing – original draft (equal). **Melanie Sal:** resources (equal), writing – original draft (equal). **Craig A. Stoops:** resources (equal), writing – original draft (equal). **Rebecca T. Trout‐Fryxell:** resources (equal), writing – original draft (equal). **Wes Watson:** resources (equal), writing – original draft (equal). **Nicole E. Wagner:** methodology (equal), resources (equal), writing – original draft (equal). **Aihua Zheng:** resources (equal), writing – original draft (equal). **Perot Saelao:** funding acquisition (equal), resources (equal), writing – original draft (equal). **Dana C. Price:** conceptualization (lead), data curation (equal), formal analysis (equal), funding acquisition (lead), methodology (equal), project administration (equal), resources (equal), supervision (equal), writing – original draft (equal), writing – review and editing (equal).

## Conflicts of Interest

The authors declare no conflicts of interest.

## Supporting information


Data S1.

## Data Availability

Sequences generated in this study have been deposited to the NCBI GenBank database under accession numbers PQ380034‐PQ380096. *Benefit‐Sharing Statement*: Benefits Generated: Benefits from this research include the generation and sharing of mitogenomes as described above.
