# Supplementary material for: Multiple Introductions of the Asian Longhorned Tick (Haemaphysalis longicornis) to the United States Revealed Using Mitogenomics
Source: Ecol Evol. 2025 Apr 24;15(4):e71312. doi: 10.1002/ece3.71312 (PMC12018891; doi:10.1002/ece3.71312)

## Supporting Information

Supplementary Table 1: Source metadata for parthenogenetic specimens.

| Sequence ID | *cox1* Haplotype | Country | State/Prefecture /Province | County (US) | Year | Host | Accession Number |
|---|---|---|---|---|---|---|---|
| SCWDS25_NSW_Australia | H3 | Australia | New South Wales | | 2013 | Horse | PQ380074.1 |
| HLP2 Hebei China | H2 | China | Hebei | | 2018 | Colony | PQ380050.1 |
| Hl-F1 Hebei China | Bisexual | China | Hebei | | 2018 | Colony | PQ380095.1 |
| Green5 Tokyo Japan | H3 | Japan | Tokyo | | 2018 | Environmental | PQ380091.1 |
| Green4 Tokyo Japan | H3 | Japan | Tokyo | | 2018 | Environmental | PQ380090.1 |
| Green8 Tokyo Japan | H3 | Japan | Tokyo | | 2019 | Environmental | PQ380088.1 |
| Green7 Tokyo Japan | H3 | Japan | Tokyo | | 2019 | Environmental | PQ380087.1 |
| Green3 Tokyo Japan | H3 | Japan | Tokyo | | 2018 | Environmental | PQ380075.1 |
| Green12 Tokyo Japan | H2 | Japan | Tokyo | | 2018 | Environmental | PQ380052.1 |
| Green6 Tokyo Japan | H2 | Japan | Tokyo | | 2019 | Environmental | PQ380093.1 |
| K15-26 Gyeonggi ROK | H3 | ROK | Gyeonggi | | 2015 | Environmental | PQ380089.1 |
| K15 Gyeonggi ROK | H3 | ROK | Gyeonggi | | 2015 | Environmental | PQ380086.1 |
| Jeju10 Jeju ROK | H3 | ROK | Jeju | | 2016 | Environmental | PQ380092.1 |
| Jeju9 Jeju ROK | Bisexual | ROK | Jeju | | 2016 | Environmental | PQ380096.1 |
| HL-AR Benton AR USA | H2 | USA | AR | Benton | 2018 | Dog | PQ380058.1 |
| DEL1 New Castle DE USA | H1 | USA | DE | New Castle | 2019 | Environmental | PQ380047.1 |
| DEL2 New Castle DE USA | H1 | USA | DE | New Castle | 2019 | Environmental | PQ380035.1 |
| DEL3 New Castle DE USA | H1 | USA | DE | New Castle | 2019 | Environmental | PQ380039.1 |
| Del4 New Castle DE USA | H3 | USA | DE | New Castle | 2019 | Environmental | PQ380077.1 |
| NC1 Surry NC USA | H2 | USA | NC | Surry | 2021 | Environmental | PQ380059.1 |
| NC2 Surry NC USA | H2 | USA | NC | Surry | 2021 | Environmental | PQ380065.1 |
| BergP8 Bergen NJ USA | H3 | USA | NJ | Bergen | 2018 | Environmental | PQ380080.1 |
| BergP13 Bergen NJ USA | H3 | USA | NJ | Bergen | 2018 | Environmental | PQ380082.1 |
| BergP17 Bergen NJ USA | H3 | USA | NJ | Bergen | 2018 | Environmental | PQ380081.1 |
| BergP24 Bergen NJ USA | H3 | USA | NJ | Bergen | 2018 | Environmental | PQ380076.1 |
| HLA1-1 Hunterdon NJ USA | H1 | USA | NJ | Hunterdon | 2017 | Sheep | PQ380038.1 |
| OrigFlem Hunterdon NJ USA | H1 | USA | NJ | Hunterdon | 2017 | Sheep | PQ380049.1 |
| HL18N1 Hunterdon NJ USA | H1 | USA | NJ | Hunterdon | 2018 | Environmental | PQ380044.1 |
| HLN9 Hunterdon NJ USA | H1 | USA | NJ | Hunterdon | 2019 | Sheep | PQ380041.1 |
| HHF7 Hunterdon NJ USA | H1 | USA | NJ | Hunterdon | 2020 | Environmental | PQ380034.1 |
| HHF32 Hunterdon NJ USA | H1 | USA | NJ | Hunterdon | 2020 | Environmental | PQ380042.1 |
| RUHL1 Middlesex NJ USA | H1 | USA | NJ | Middlesex | 2018 | Environmental | PQ380037.1 |
| RUHL2 Middlesex NJ USA | H1 | USA | NJ | Middlesex | 2018 | Environmental | PQ380036.1 |
| SOM2 Somerset NJ USA | H1 | USA | NJ | Somerset | 2019 | Dog | PQ380040.1 |

| | | | | | | | |
|---|---|---|---|---|---|---|---|
| BbNY1 Somerset NJ USA | H1 | USA | NJ | Somerset | 2020 | Environmental | PQ380045.1 |
| BbAD Somerset NJ USA | H1 | USA | NJ | Somerset | 2020 | Environmental | PQ380046.1 |
| DVHL1 Union NJ USA | H1 | USA | NJ | Union | 2018 | Environmental | PQ380048.1 |
| DVHL2 Union NJ USA | H2 | USA | NJ | Union | 2018 | Environmental | PQ380072.1 |
| Yonk1 Westchester NY USA | H3 | USA | NY | Westchester | 2018 | Environmental | PQ380083.1 |
| HL12 Suffolk NY USA | H3 | USA | NY | Suffolk | 2019 | Canada goose | PQ380079.1 |
| MPA4-9 Chester PA USA | H1 | USA | PA | Chester | 2019 | Chicken | PQ380043.1 |
| Rlad Newport RI USA | H3 | USA | RI | Newport | 2023 | Environmental | PQ380078.1 |
| Cocke TN USA | H2 | USA | TN | Cocke | 2021 | Environmental | PQ380051.1 |
| Roane TN USA | H2 | USA | TN | Roane | 2021 | Environmental | PQ380070.1 |
| Union TN USA | H2 | USA | TN | Union | 2021 | Environmental | PQ380062.1 |
| SCWDS1 Albemarle VA USA | H2 | USA | VA | Albemarle | 2018 | White-tailed deer | PQ380073.1 |
| SCWDS16 Augusta VA USA | H2 | USA | VA | Augusta | 2018 | White-tailed deer | PQ380054.1 |
| Montgomery VA USA | H2 | USA | VA | Montgomery | 2021 | Environmental | PQ380060.1 |
| Pulaski VA USA | H2 | USA | VA | Pulaski | 2021 | Environmental | PQ380053.1 |
| SCWDS5 Staunton VA USA | H2 | USA | VA | Staunton | 2018 | White-tailed deer | PQ380061.1 |
| Wythe VA USA | H2 | USA | VA | Wythe | 2021 | Environmental | PQ380068.1 |
| WV16 Hardy WV USA | H2 | USA | WV | Hardy | 2018 | Environmental | PQ380064.1 |
| WV2 Mason WV USA | H2 | USA | WV | Mason | 2018 | Dog | PQ380063.1 |
| WV8 Mason WV USA | H2 | USA | WV | Mason | 2018 | Dog | PQ380071.1 |
| NWV1 Ritchie WV USA | H2 | USA | WV | Ritchie | 2019 | Cattle | PQ380055.1 |
| NWV2 Ritchie WV USA | H2 | USA | WV | Ritchie | 2019 | Cattle | PQ380057.1 |
| SCWDS2 Upshur WV USA | H3 | USA | WV | Upshur | 2018 | Coyote | PQ380085.1 |
| WV332 Upshur WV USA | H2 | USA | WV | Upshur | Unknown | Unknown | PQ380066.1 |
| WV333 Upshur WV USA | H3 | USA | WV | Upshur | Unknown | Unknown | PQ380084.1 |
| WV335 Upshur WV USA | H2 | USA | WV | Upshur | Unknown | Unknown | PQ380069.1 |
| WV340 Upshur WV USA | H2 | USA | WV | Upshur | Unknown | Unknown | PQ380056.1 |
| WV341 Upshur WV USA | H2 | USA | WV | Upshur | Unknown | Unknown | PQ380067.1 |

Supplementary Table 2: List of non-synonymous substitutions in genes of parthenogenetic specimens belonging to clades H1, H2, and H3. Those substitutions identified as driving significant selective pressure analysis results are indicated in **bold**. Note that all SNPs are based on comparison to the reference genome at GenBank Accession number MG450553.1.

| Gene | Nucleotide Change | Amino Acid Change | Lineage |
|---|---|---|---|
| *cox1* | T322A | L>M | H1, H2, H3 |
| | C583A | L>M | H1, H3 |
| | A1219G | I>V | H1, H2, H3 |
| *cox3* | G79A | G>S | H1, H2, H3 |
| | G461A | S>N | H1, H2, H3 |
| | G665T | S>M | H1, H3 |
| *cytb* | C7T | L>F | H1, H2, H3 |
| | A61G | M>V | H1, H3 |
| | T911C | L>S | H1, H2, H3 |
| | T1008C | F>S | H1, H2, H3 |
| *nad1* | A336G | I>M | H1, H2, H3 |
| | A519G | X>W | H1, H2, H3 |
| | A520G | I>V | H1, H2, H3 |
| | A726G | I>M | H1, H2, H3 |
| | G766A | V>I | H1, H2, H3 |
| | **T944A** | **F>Y** | **H1** |
| *nad2* | C419T | A>V | H1, H2, H3 |
| | T431A | F>Y | H1, H2, H3 |
| | A454G | I>V | H2 |
| | A619G | M>V | H1, H2, H3 |
| *nad3* | G5T | C>F | H2 |
| *nad4* | A111T | L>F | H1, H2, H3 |
| | A116T | Y>F | H1, H3 |
| | C327A | F>L | H1, H2, H3 |
| | G486A | M>I | H1, H2, H3 |
| | C509T | S>L | H1, H2, H3 |
| | C1083A | F>L | H1, H2, H3 |
| | G1173A | M>I | H1, H2, H3 |
| *nad4l* | G31A | V>I | H1, H2, H3 |
| | **G115A** | **V>M** | **H1, H2, H3** |
| *nad5* | C115A | L>M | H1, H2, H3 |
| | T777A | F>L | H1, H2, H3 |
| | T1031A | F>Y | H1, H2, H3 |
| | T1153C | F>L | H2, H3 |
| | A12676 | I>V | H1, H2, H3 |
| | C1367A | A>V | H1, H3 |
| | T1403A | L>Q | H1, H2, H3 |
| *nad6* | C76A | L>I | H1, H2, H3 |
| | C272T | S>L | H1, H2, H3 |
| *atp6* | T65G, G66A | L>W | H1, H2, H3 |
| | A223G | I>V | H1, H2, H3 |
| | T264A | I>M | H1, H2, H3 |
| | C514T | H>Y | H2 |
| | G577A | V>I | H1, H2, H3 |
| | A580G | M>V | H1, H2, H3 |
| *atp8* | C28A | L>M | H3 |
| | A49G | M>V | H1, H2, H3 |
| Reference: MG450553.1 | | | |

Supplementary Figure 1: Maximum-likelihood phylogeny corresponding to the monophyletic H1 clade. Branch lengths are proportional to the number of substitutions per site. Note that all H1 sourced from the US are identical at the sequence level, though small gaps in sequencing coverage create some phylogenetic signal.

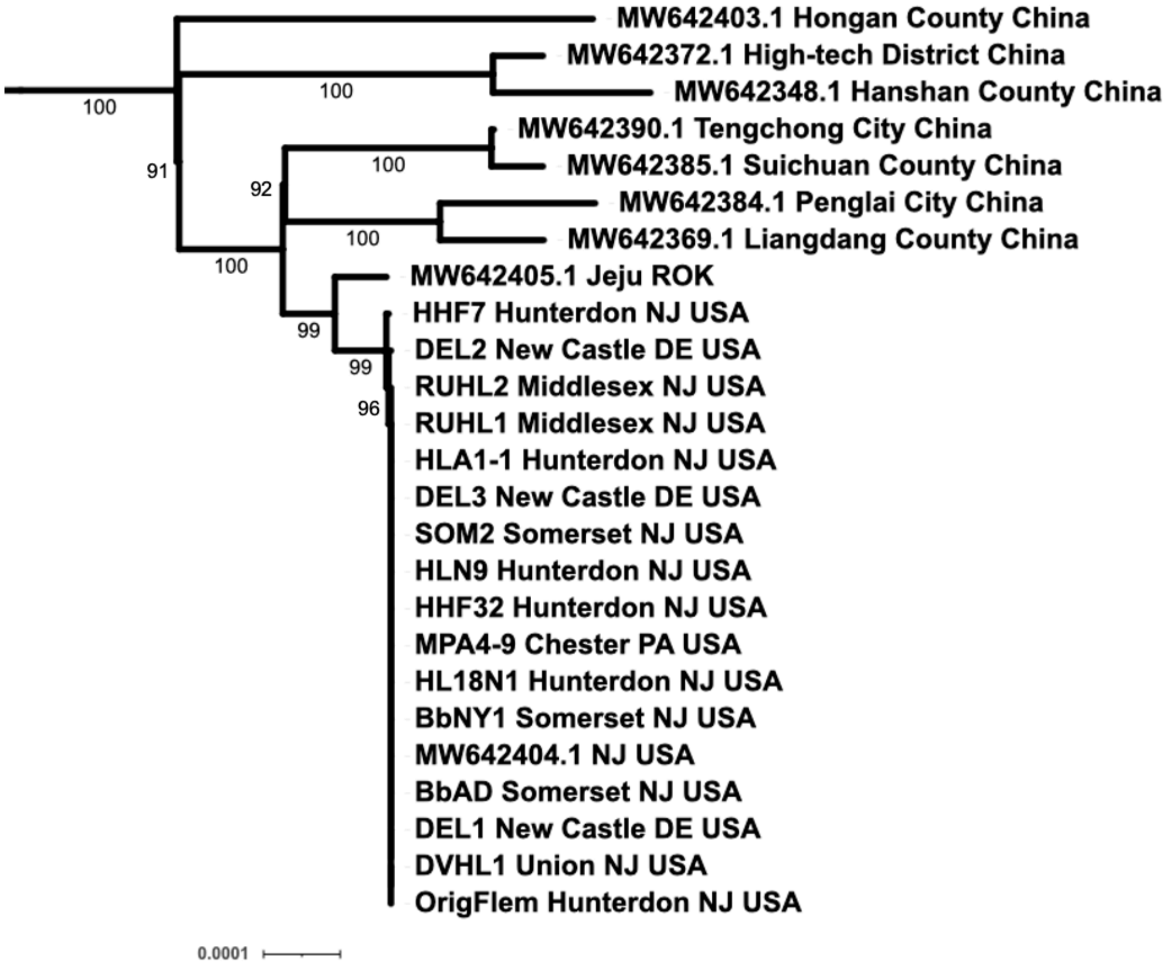

Supplementary Figure 2: Maximum-likelihood phylogeny corresponding to the monophyletic H2 clade. Branch lengths are proportional to the number of substitutions per site.

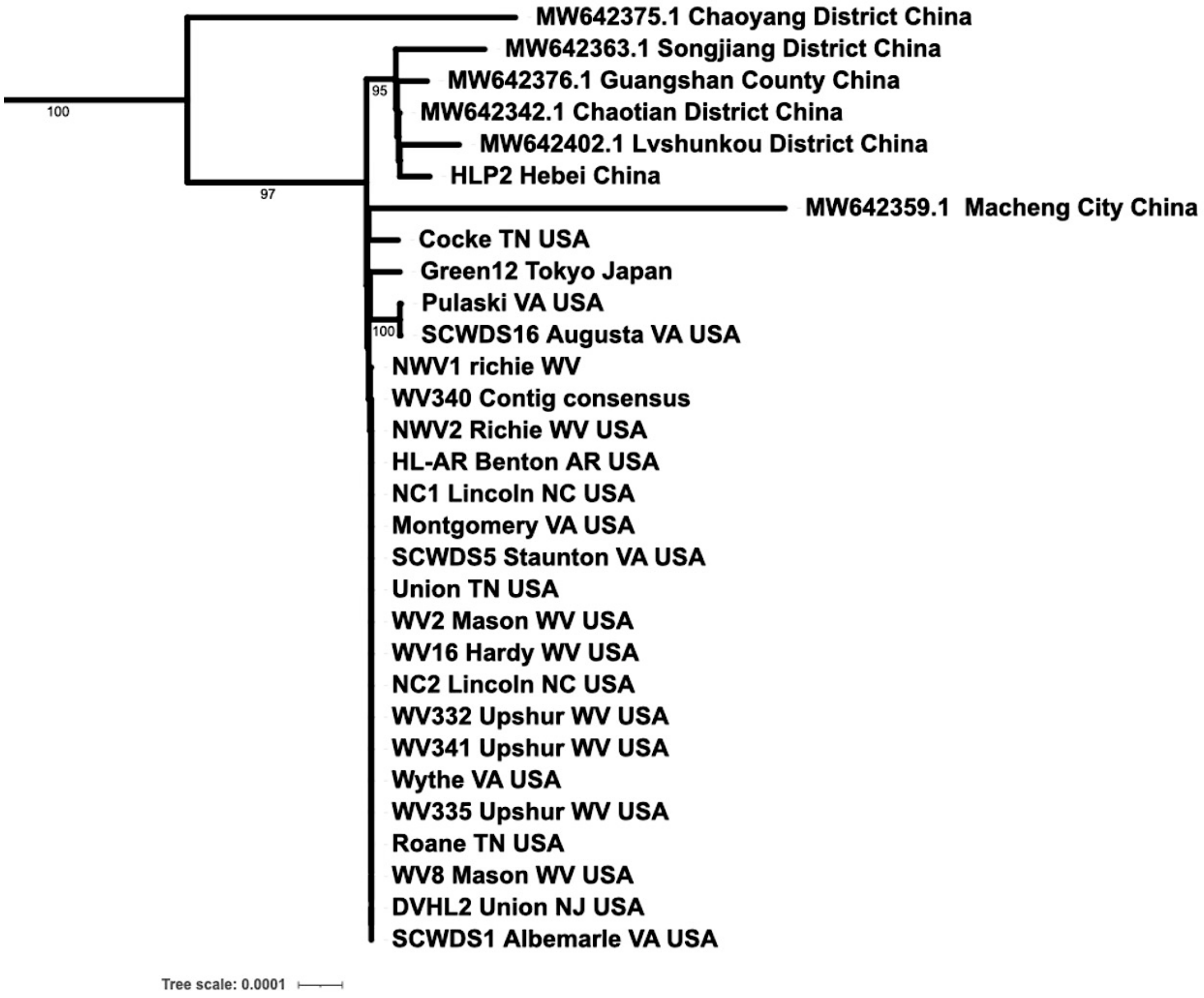

Supplementary Figure 3: Maximum-likelihood phylogeny corresponding to the monophyletic H3 clade. Branch lengths are proportional to the number of substitutions per site.

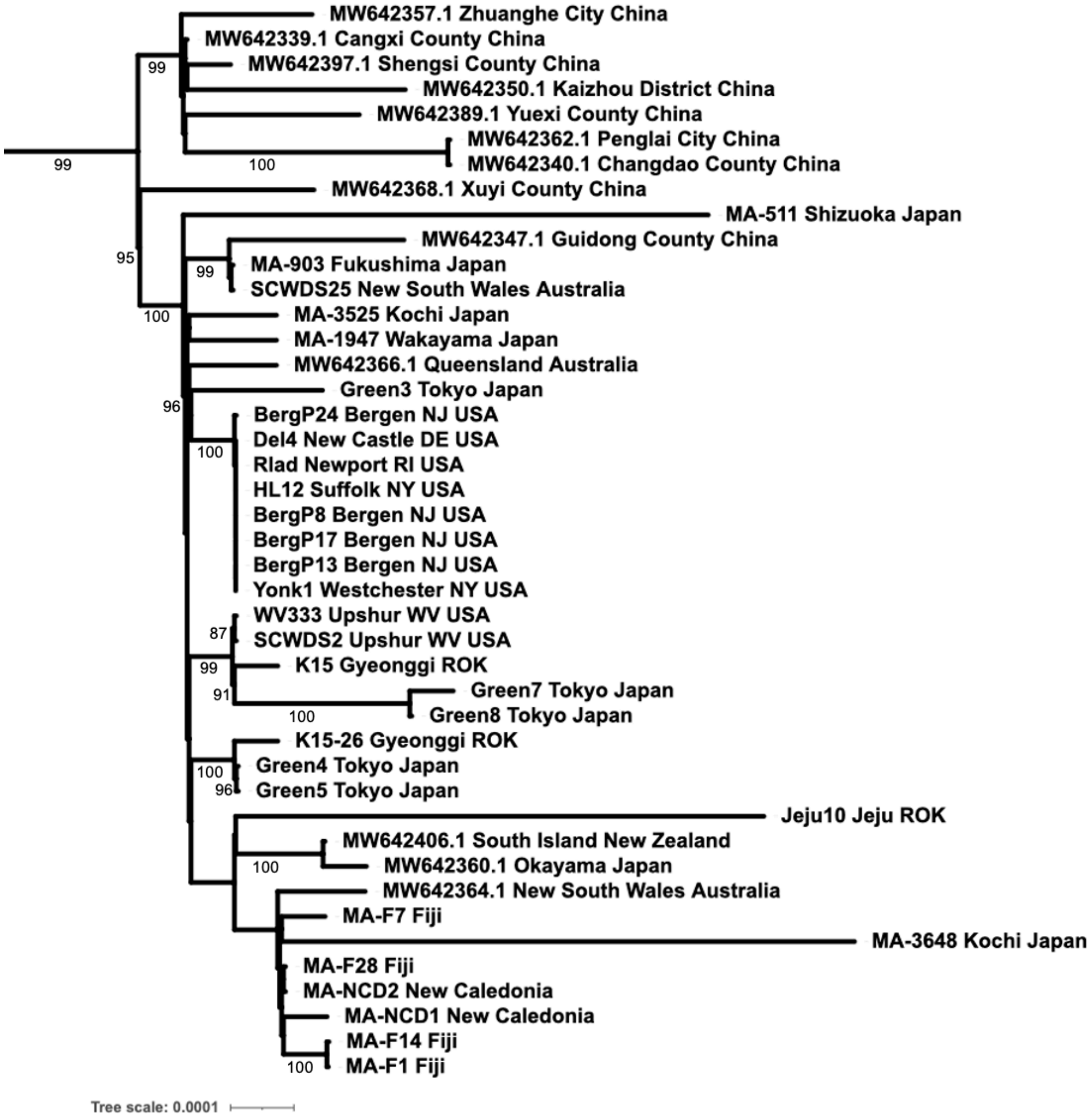

Supplement: Supplementary file 1 — Data S1. [file ECE3-15-e71312-s001.pdf]
